# Harnessing the secretome of adipose-derived stem cells in the treatment of ischemic heart diseases

**DOI:** 10.1186/s13287-019-1289-7

**Published:** 2019-06-27

**Authors:** Xiaoting Li, Teng Ma, Jiacheng Sun, Mingjing Shen, Xiang Xue, Yongbing Chen, Zhiwei Zhang

**Affiliations:** 1 Department of Cardiothoracic Surgery, The Second Affiliated Hospital of Soochow University, No.1055, Sanxiang Road, Suzhou, 215004 China; 2 Department of Cardiology, The Second Affiliated Hospital of Soochow University, No.1055, Sanxiang Road, Suzhou, 215004 China; 3 Department of Cardiovascular Surgery, The First Affiliated Hospital & Institute for Cardiovascular Science, Soochow University, No.899, Pinghai Road, Suzhou, 215006 China

**Keywords:** Adipose-derived stem cells, Ischemic heart diseases, Paracrine effects, Secretome, Myocardial repair, Strategies

## Abstract

Adipose-derived stem cells (ASCs) are promising therapeutic cells for ischemic heart diseases, due to the ease and efficiency of acquisition, the potential of myocardial lineage differentiation, and the paracrine effects. Recently, many researchers have claimed that the ASC-based myocardial repair is mainly attributed to its paracrine effects, including the anti-apoptosis, pro-angiogenesis, anti-inflammation effects, and the inhibition of fibrosis, rather than the direct differentiation into cardiovascular lineage cells. However, the usage of ASCs comes with the problems of low cardiac retention and survival after transplantation, like other stem cells, which compromises the effectiveness of the therapy. To overcome these drawbacks, researchers have proposed various strategies for improving survival rate and ensuring sustained paracrine secretion. They also investigated the safety and efficacy of phase I and II clinical trials of ASC-based therapy for cardiovascular diseases. In this review, we will discuss the characterization and paracrine effects of ASCs on myocardial repair, followed by the strategies for stimulating the paracrine secretion of ASCs, and finally their clinical usage.

## Introduction

Ischemic heart diseases (IHD), specifically acute myocardial infarction (AMI), are still the primary causes of morbidity and mortality globally [1], in spite of the great improvements in medical and surgical therapy. Cardiac transplantation seems to be the last resort available for replacing the terminally exhausted heart. However, the number of donors is limited. Fortunately, stem cell-based therapy provides a promising and burgeoning option for the treatment of impaired myocardial tissue in the twenty-first century. Stem cell transplantation has been well investigated and showed significant effects on cardiac repair, by reducing left ventricle (LV) remodeling and improving cardiac function after MI [2]. The therapeutic stem cells include bone marrow-derived mesenchymal stem cells (BMSCs), adipose-derived stem cells (ASCs), endothelial progenitor cells (EPCs), embryonic stem cells (ESCs), induced pluripotent stem cells (iPS cells), and other emerging therapeutic stem cell types for IHD [3]. Nevertheless, ASCs exhibit much more attractive advantages in the treatment of impaired myocardium compared with other stem cell types, owing to the ease and high efficiency of acquisition, the potential of rapid proliferation, and the advantageous properties of myocardial lineage differentiation potential and immune tolerance [4–6].

Numerous experimental and clinical studies have indicated that ASC-based therapy of IHD is surprisingly effective in the restoration of impaired cardiac function [7–9]. However, the exact repair mechanisms still remain controversial. Interestingly, accumulating evidences have shown that the rate of survival and cardiac retention of stem cells is extraordinarily low after transplantation, even though a remarkable improvement of cardiac function has been observed [7, 10]. Therefore, researchers speculated that the great benefits were predominantly attributed to the paracrine effects rather than the direct differentiation into cardiovascular lineage cells. This hypothesis had been well verified in BMSCs [11, 12] and ASCs [13]. Yang et al. [14] revealed that ASC-derived vascular cells only amount to about 9% of the enhanced angiogenesis after implantation for MI and further confirmed that the paracrine effects of ASCs played a major role underlying cardiac protective function. In fact, the exact paracrine function of ASCs probably depends on the trophic factors secreted by ASCs, including cytokines, growth factors, extracellular microvesicles or exosomes, and micromolecular materials, broadly termed as the secretome [12].

Here, we introduce the current understanding of the ASC secretome and their functional roles in cardiac repair and subsequently discuss in detail the current strategies to stimulate the release of the ASC secretome. Finally, we highlight the clinical application of ASCs for IHD.

## Characterization of ASCs and profiling their secretome

Zuk et al. [15] first found a population of stem cells, which were isolated from human processed lipoaspirate, presented multilineage potential similar to that of bone marrow-derived MSCs and were termed as ASCs later on. BMSCs were first discovered and regarded as the primary source of stromal stem cells for clinical application. Later on, further studies identified that ASCs were even superior to BMSCs because of the ease of isolation, the safety of investigation, and the considerably large amounts available, which became one of the most promising alternatives to BMSCs [16]. Researchers estimated that BMSCs constituted approximately 0.001–0.01% of the total marrow nucleated cells, whereas the amount of ASCs was much higher than that of BMSCs when isolated from equivalent amounts of adipose and bone marrow tissue [16]. Fraser et al. [6] compared the frequency and yield between ASCs and BMSCs by applying clonogenic assays for either fibroblastoid-like colonies (CFU-F) or colonies expressing alkaline phosphatase (CFU-AP) and found that the frequency of CFU-F and CFU-AP in adipose tissue was 500-fold more than that in marrow (ASCs: 1 in 100; BMSCs: between ~ 1 in 50,000 and 1 in 100,000). Thus, unlike BM-MSCs, freshly isolated ASCs from adipose tissue can be readily used in therapy without expansion [17].

Like other somatic stem cells, ASCs are characterized by the expression of specific surface immunophenotypes. According to previous reports, the human ASCs express characteristic surface markers including CD9, CD13, CD29, CD44, CD55, CD71, CD90, CD105, and HLA-ABC and negatively express surface markers including CD11b, CD16, CD18, CD31, CD45, CD104, and HLA-DR [18]. In addition, the phenotype of ASCs mostly resembles bone marrow-derived MSCs, such as CD13, CD29, CD44, CD58, and CD166 [19]. However, the exact expression profile of ASCs remains unclear, due to the inconsistent phenotype seen in the source of different species, the method of acquisition, the stage of cultivation, and the source of adipose tissue depot [19].

Regarding the differentiation potential, ASCs can differentiate into several cell types, including adipose, bone, cartilage, skeletal muscle, cardiac muscle, neuronal cells, hematopoietic cells, endothelial cells, and hepatocytes in the presence of lineage-specific induction factors and specific extracellular milieu [6]. Nevertheless, the differentiation for stromal stem cells might be biased toward the origin tissue [16]. Recent results suggested that ASCs presented a pronounced capacity of adipogenic differentiation compared with BMSCs in vitro [20]. Consistently, researchers [21] demonstrated that BMSCs were more prone to osteogenic differentiation than ASCs in vitro.

Notably, ASCs from different sources displayed distinct characteristics. Ong et al. [22] reported that the surface markers of subcutaneous adipose were significantly different from those of visceral adipose tissue depots. Subcutaneous adipose-derived ASCs showed increased expression of CD10, whereas ASCs from visceral adipose expressed higher levels of CD200. Also, the differentiation potential of ASCs is related to their specific anatomic locations [23]. For instance, the subcutaneous adipose-derived ASCs could differentiate better into mature adipocytes than those from visceral adipose in vitro [24]. Furthermore, Harmelen et al. [25] confirmed that ASCs from the subcutaneous adipose tissue region from obese subjects proliferated faster than those from the omental region. Compared with omental preadipocytes, human abdominal subcutaneous adipose precursors underwent extensively increased lipid accumulation, enhanced adipogenic transcription factor expression, and decreased TNF-α-induced apoptosis [26]. Additionally, the ASCs isolated from the superficial adipose layers differentiated quicker to a greater extent than those from the deep adipose layers of abdominoplasty specimens [27]. Except for the adipose tissue depot origin, donor characteristics such as age, sex, and metabolic status also affected the differentiation potential of ASCs [28].

For therapeutic IHD, ASCs display some favorable features, including directly differentiating into cardiovascular lineage cells and offering the secretome such as cytokines, growth factors, and exosomes [29]. A typical ASC secretome profile comprises various growth factors, cytokines, RNAs, and lipid mediators. Several trophic factors secreted by ASCs have been reported, such as vascular endothelial growth factor (VEGF), hepatocyte growth factor (HGF), insulin-like growth factor (IGF)-1, β-nerve growth factor (NGF), stromal cell-derived factor (SDF)-1α, and exosomes, which are functional in cardiovascular diseases therapy [30]. Through mass spectrometry analysis, Riis et al. [31] revealed that the secretome of human ASCs consisted of 342 proteins in the normoxic condition. They were related to angiogenesis and vasculature development, extracellular matrix (ECM) formation, cell adhesion/migration, cell survival/death, and immune regulation.

However, the secretome expression is heterogeneous and diverse in distinct microenvironments and disease status, such as hypoxia, serum-free, obese, and diabetes. Togliatto et al. [32] found that the paracrine factors of obese ASCs, including VEGF, matrix metalloproteinase 2 (MMP-2), and miR-126 in extracellular vesicles, were largely reduced compared with those of non-obese ASCs. Meanwhile, the secretome profile in vivo is much more complicated and dynamic compared with that in vitro. Therefore, the specific and dynamic expression profile of ASCs is a Gordian knot to be unraveled in different microenvironments. The future study should aim at quantifying the secreting factors in the secretome profile of ASCs in vivo.

## Functions of the ASC secretome in myocardial repair

**Fig. 1:**
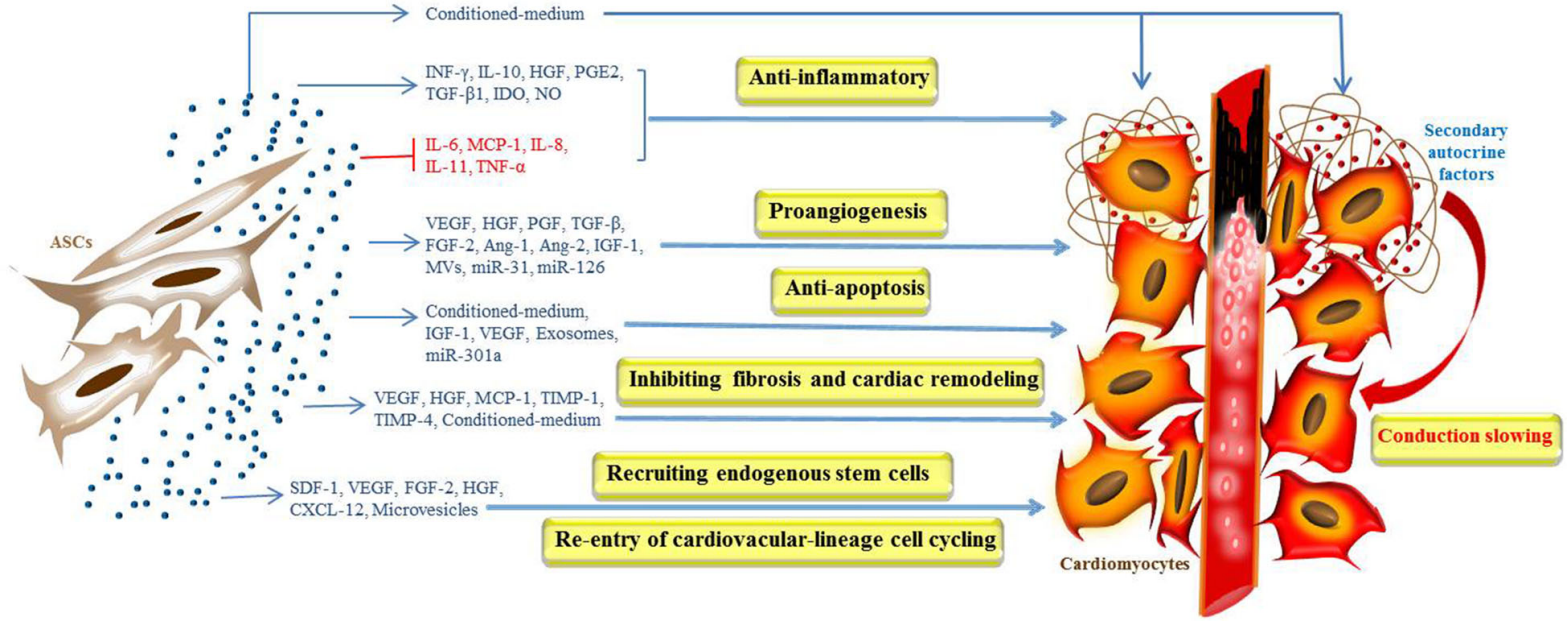
Functions of the ASC secretome on the ischemic heart. Lots of trophic factors released by ASCs, such as VEGF, HGF, PGF, TGF-β, FGF-2, Ang-1, and Ang-2, IGF-1, and microvesicles, miR-31 and miR-126, benefit for proangiogenesis in the ischemic myocardium. The ASC secretome may have the ability to modulate the release of inflammatory cytokines, such as INF-γ, IDO, NO, IL-6, IL-8, IL-10, IL-11, and TNF-α. ASC-secreted factors such as IGF-1, VEGF, exosomes, and miR-301a may improve the capacity of cardiomyocyte survival in hypoxic conditions. Meanwhile, ASCs may secrete molecules such as VEGF, HGF, MCP-1, TIMP-1, and TIMP-4 that contribute to inhibiting fibrosis and cardiac remodeling. Maybe certain paracrine factors such as SDF-1, VEGF, FGF-2, HGF, CXCL-12, and microvesicles released by ASCs could recruit endogenous stem cells and enable cardiovacular lineage cells re-enter cell cycling. In addition, ASC-conditioned medium could induce conduction slowing of neonatal rat ventricular myocytes (NRVMs), probably attributed to the secondary autocrine myocardial factors released by NRVM

**Fig. 2:**
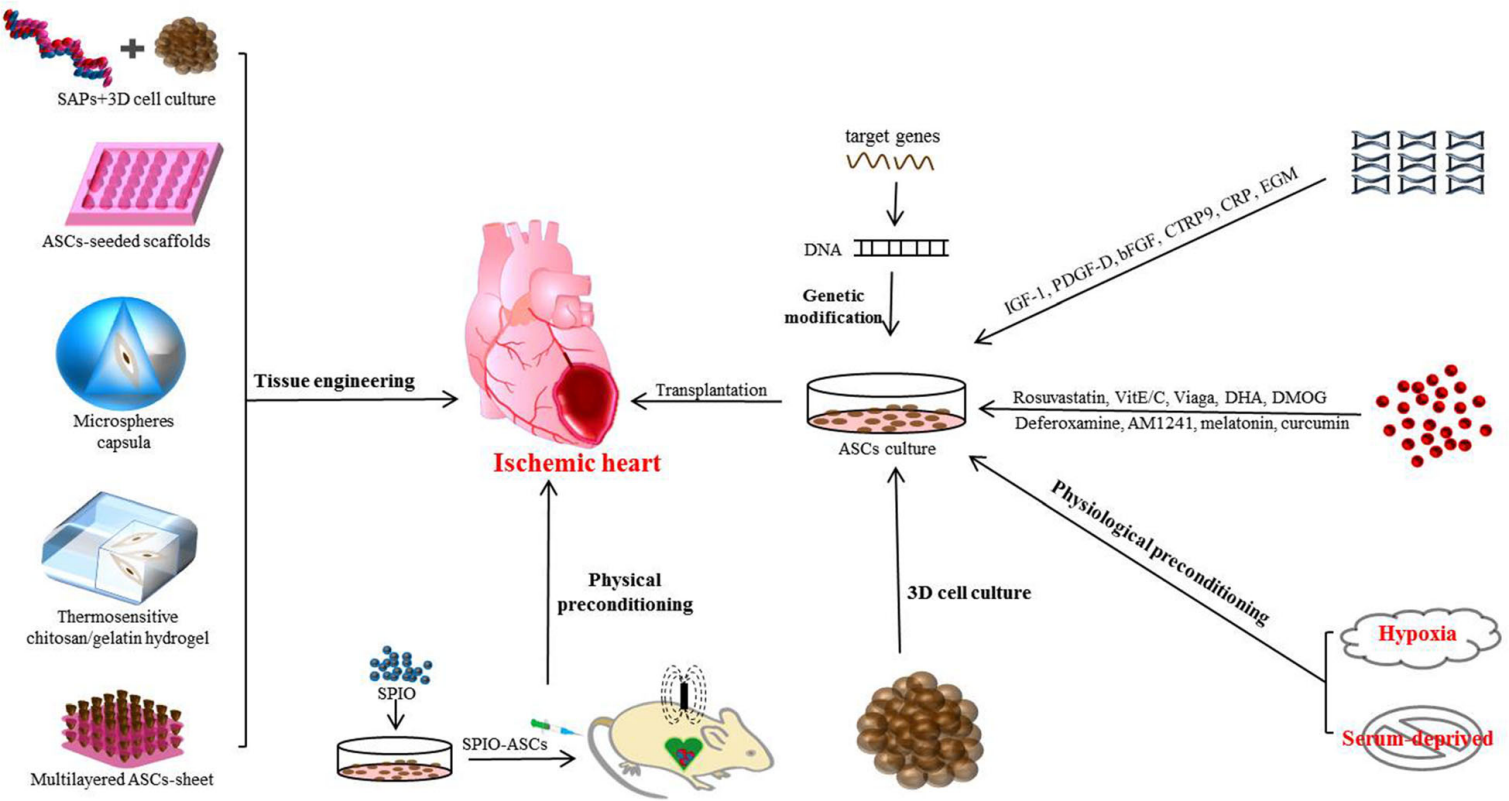
Paracrine effects of the ASC secretome and involved pathways. ASCs secrete IGF-1, SOD-3, miRNA-301a, and exosomes for improving the effect of anti-apoptosis or pro-survival through regulating cell cycle, N-Cadherin/ERK/Nrf2, ASK-1 and NFγB/p38/JNK, Wnt/β-Catenin, cyclin D1, Bcl-2 and Bax, respectively. ASC-derived microvesicles perform the pro-angiogenesis effect via delivery of miRNA-126 and miRNA-31 through ERK1/2/MAPK and FIH-1 pathways, respectively. The conditioned medium of ASCs exerts the anti-inflammation effect through IFN-γ/IDO and PGE_2_/EP2/4 signaling pathways, which also can recruit circulating EPCs through CXCL12/SDF-1α pathway. The anti-cardiac remodeling effect is mediated by exosomes through MAPK/PI3K pathway and HGF released from ASCs

The extensive researches on mechanisms underlying ASC-based therapy for IHD in the past decade have unveiled some of the key functional roles that ASC secretome play in the restoration of impaired cardiomyocytes and improvement of cardiac function [30]. The functions of ASC secretome for cardiac repair are briefly summarized in Fig. 1. And the paracrine factors of ASCs and related pathways are summarized in Fig. 2.

### Anti-apoptosis

After myocardial infarction, a mass of cardiomyocytes undergo the pathological process of apoptosis due to ischemia and hypoxia. Fortunately, using the ASC secretome-based therapy can largely prevent this adverse outcome. Chen et al. [33] showed that the ASC-conditioned medium (ASC-CM) could notably mitigate the apoptosis of cardiomyocytes induced by porphyromonas gingivalis endotoxin (Pg-LPS), along with the change of the expression of apoptosis-related factors including t-Bid and caspase-3. Yang et al. [14] also observed that in the MI border zone, the apoptosis of cardiomyocytes was significantly reduced after the transplantation of ASC-CM. Furthermore, through hypoxic induction, the release of VEGF was significantly increased in ASC-CM, which exerted the effects of pro-survival and anti-apoptosis on endothelial cell [34]. Besides ASC-CM, ASC-derived exosomes (ASC-exo) also show the specific repair function of damaged cardiomyocytes. Exosomes, secreted by most cell types, are lipid bilayer membrane structures with a diameter of about 30–100 nm [12]. They acted as the carrier to mediate the communication between ASCs and damaged cardiomyocytes by shuttling various micromolecular materials such as proteins and RNAs. Also, ASC-exo could reverse ischemia- or hypoxia-induced myocardial apoptosis. After hypoxia/reoxygenation-induced myocardial injury, ASC-exo significantly regulated the expression of apoptosis-related markers, such as Bax, Bcl-2, and Cyclin D1, through the Wnt/β-catenin signaling pathway [35]. In a variety of delivery RNAs, Lee et al. [36] confirmed that miRNA-301a enriched in human ASCs could significantly attenuate apoptosis of injured cardiomyocytes by inhibiting the apoptosis signal-regulating kinase 1 (ASK1), which regulated the downstream of p38/JNK/NFγB signaling pathways.

### Proangiogenesis

Many researches have reported that the ASC secretome contributes to the angiogenesis in the ischemic heart. Among lots of cytokines, the angiogenesis-related cytokines secreted by ASCs, such as HGF, VEGF, placental growth factor (PGF), transforming growth factor (TGF)-β, fibroblast growth factor (FGF)-2, angiopoietin (Ang)-1, and Ang-2, are able to directly act on endothelial cells and thus increase their pro-angiogenesis capacity [37]. Rehman et al. [34] also observed that the pro-angiogenic potential of ASCs appeared following the secretion of VEGF, HGF, and TGF-β. Additionally, the microvesicles (MVs) released by ASCs, playing a vital role in intercellular communication, also have the potential of pro-angiogenesis. Kang et al. [38] delivered microRNA-31 from MVs to endothelial cells (ECs), targeting the factor-inhibiting HIF-1 (FIH-1), and found that MVs could promote the migration and vascular formation of ECs. Togliatto et al. [32] also found that miRNA-126 in ASC-derived MVs could prompt angiogenesis by activating the extracellular signal-regulated protein kinase 1/2/mitogen-activated protein kinase (Erk1/2/MAPK) pathway in patients with diabetes mellitus. Regarding the ASC-based therapy for AMI, Wang et al. [39] demonstrated that the capillary density in the infarct border zone was significantly augmented, resulting from the cardiac protective growth factors secreted by ASCs, such as VEGF, HGF, and IGF-1, although only 0.5% of the recovered ASCs stained positive for cardiac-specific fibril proteins. In addition, Oliva-Olivera et al. [40] delineated an interesting phenomenon that the level of VEGF in the conditioned medium secreted by ASCs derived from the thymus (thymASCs) was remarkably higher than that from subcutaneous adipose (subASCs). This explains the stronger pro-angiogenic effect of thymASCs than that of subASCs.

### Anti-inflammation

The inflammation is one of the crucial pathophysiological processes after AMI and may lead to poor prognosis. ASCs possess the potential to regulate inflammatory stress through secreting various inflammatory-related factors. Olga et al. [41] illustrated that conditioned supernatants of human ASCs had an obvious impact on the immunization of peripheral blood mononuclear cells, accompanied by the release of immunosuppressive soluble factors including interferon (IFN)-γ, IL-10, HGF, prostaglandin E_2_ (PGE_2_), TGF-β1, indoleamine 2,3-dioxygenase (IDO), and nitric oxide. The IFN-γ/IDO axis might be the essential pathway in the anti-inflammation process. Hao et al. [42] also found that the PGE_2_ secreted by ASCs were anti-inflammatory, due to the enhanced release of IL-10 by the polarization of activated M2 macrophages through the PGE_2_-EP2/4 axis. Regarding the immunoregulation of the ASC secretome on myocardium repair, Kim et al. [43] confirmed that the circulating pro-inflammatory cytokine levels of IL-6 and monocyte chemoattractant protein (MCP)-1 were dramatically decreased after transplantation of mASCs^hTERT^ (the human telomerase reverse transcriptase gene transfected into mouse ASCs) for AMI therapy, which were remarkably decreased after the application of CD34^−^mASCs^hTERT^. Future studies should put more effort to examine the significant anti-inflammatory effect of ASC secretome in the therapy of IHD.

### Inhibition of fibrosis and cardiac remodeling

MI is frequently accompanied by cardiac fibrosis and remodeling, consequently leading to the development of ventricular dilatation and congestive heart failure. Nevertheless, the transplantation of ASCs provides a unique therapeutic strategy to reverse fibrosis and prevent the impaired heart from adverse matrix remodeling. The remodeling process involves the modulation of various components of ECM and is mediated by the paracrine effects of ASCs. Mazo et al. [44] reported that after the transplantation of ASCs for ischemia/reperfusion-induced swine cardiac injury, the cardiac fibrosis and the ventricular impairment were dramatically improved in 3 months. Although the engraftment of ASCs was not observed, the ratio of MMP/TIMP was changed, which strongly suggested that the paracrine effect may account for the cardiac repair and remodeling. Furthermore, Li et al. [45] had manifested that HGF was a major effective contributor for anti-fibrosis function of ASCs.

Wang et al. [39] also found that the paracrine effect of ASCs may be responsible for the improvement of cardiac function and dilation, as only 0.5% of the transplanted ASCs were retained in the ASC-treated heart. Furthermore, Yang et al. [14] confirmed that ASC-CM treatment alone, depending on various trophic cardioprotective factors secreted by ASCs in the medium, was sufficient to reduce myocardial infarct size and improve cardiac function for MI mice. To further determine the cardioprotective components in the ASC-CM, they reported that trophic factors, including VEGF, HGF, MCP-1, TIMP-1, and TIMP-4, were beneficial for reducing scar fibrosis and inhibiting cardiac hypertrophy and remodeling for MI therapy [46]. In addition, ASC-derived exosomes could significantly decrease intimal thickness through activation of MAPK/PI3K signaling pathways, contributing to anti-vascular remodeling [47].

### Recruitment of endogenous stem cells and re-entry of cardiovascular lineage cell cycling

Through the paracrine effect, the transplanted ASCs should have the capacity of mobilizing and recruiting autologous stem cells, which reside in the adult tissue or organs such as the peripheral blood, bone marrow, and adipose depot [2, 48]. It was reported that after the transplantation of ASCs, the chemotactic factor of SDF-1 in the blood was increased, and circulating EPCs migrated to the transplanted region and were enriched in the ischemic issue [49]. In addition, in order to improve the efficiency of recruitment, Bhang et al. [50] utilized the technique of three-dimensional (3D) spheroid cultivation to facilitate ASCs releasing much more paracrine factors, such as VEGF, FGF-2, HGF, and chemokine (C-X-C motif) ligand (CXCL)-12. These factors could promote EPC mobilization from the bone marrow and homing to the ischemic location.

Hatzistergos et al. [51] reported that intra-myocardial injection of BMSCs could mobilize host endogenous c-kit^+^ stem cells, but injection of BMSC-conditioned medium alone could not achieve the same effect. BMSC delivery was likely to provide enormous advantages for the long-standing retention and persistent releasing of paracrine factors from the cells, which served as the basis of cardiac repair. Furthermore, they found that the mitotic cardiomyocytes distinctly increased, indicating the stimulation of endogenous cardiomyocytes and re-entry of cell cycling.

Based on the mechanism of BMSC-based therapy for cardiac repair, we hypothesize that ASCs may also be able to recruit endogenous stem cells to the damaged cardiac region and stimulate cardiomyocyte to re-enter cell cycling through paracrine action. More investigations are still required to further establish the role of paracrine in the functionality of ASCs.

## The impact on cardiac electrophysiology

Although the ASC secretome presents favorable effects in cardiac repair, the potential pro-arrhythmic influence is non-negligible. However, currently, little is known how ASC secretome affects cardiac electrophysiology. It was reported [52] that porcine ASC-conditioned medium significantly reduced the conduction velocity of neonatal rat ventricular myocytes (NRVMs) measured by electrical mapping and microelectrode recordings. The secretome of porcine ASCs (pASCs) impaired cardiomyocytes, leading to an increased electrophysiological heterogeneity and conduction dysfunction. Smit et al. [53] further explored the underlying mechanism of the potential pro-arrhythmic impact of pASC secretome. They demonstrated that the secretome could induce NRVM to release the secondary autocrine myocardial factors, which were responsible for the cardiac electrophysiological change. Consistently, using a kind of biomaterial, recombinant human collagen-based microspheres, the induced secondary autocrine effect of NRVM was significantly reduced, and finally, the cardiac conduction slowing was notably mitigated. The effects of ASC secretome on cardiac electrophysiology were briefly summarized in Fig. 1.

## Strategies for stimulating ASC paracrine secretion

**Fig. 3:**
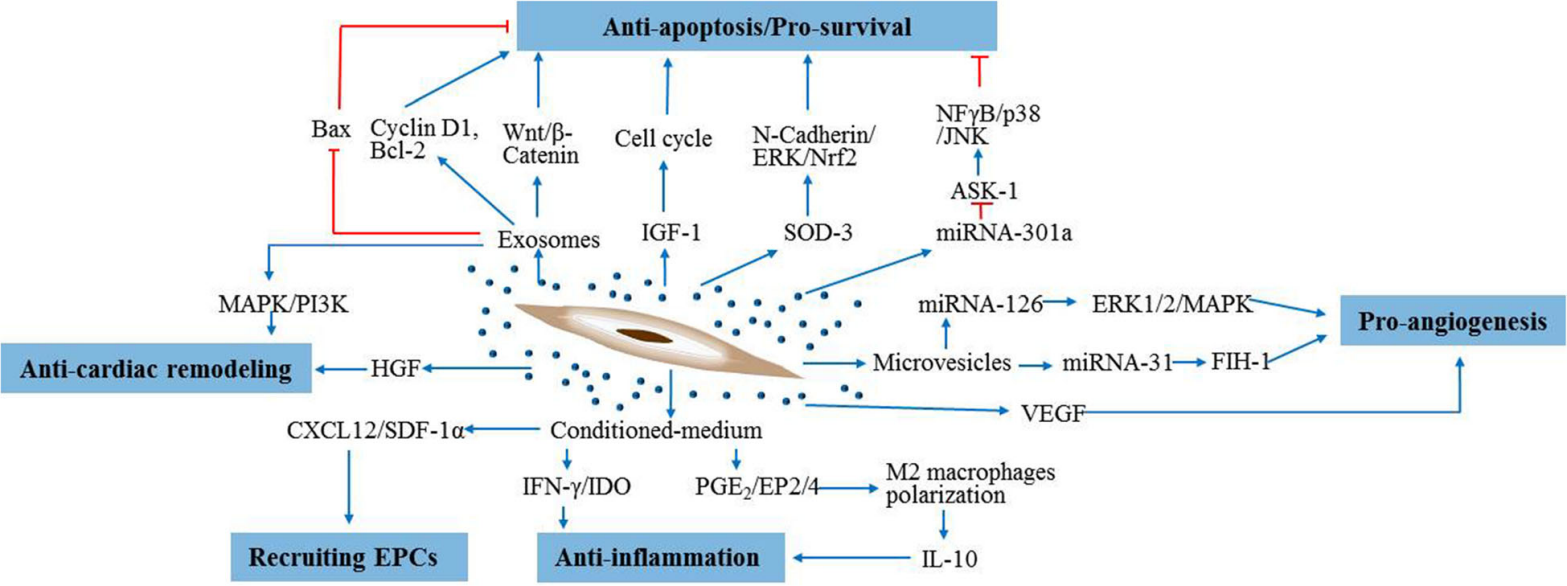
Strategies for stimulating ASC paracrine secretion are summarized. Genetic modification and protein molecular, pharmacological, physiological, and physical preconditioning have been employed to stimulate the release of the customized ASC secretome. Furthermore, bioengineering strategies, such as 3D cell culture combined with SAPs, ASC-seeded scaffolds, microspheres capsula, thermosensitive chitosan/gelatin hydrogel, and cell sheet, can efficiently control and maintain the release of ASC secretome through several smart biomaterials

A major issue of the usage of implanted ASCs in the treatment of IHD is the low cardiac retention rate. Also, the concentration and volume of paracrine factors released by ASCs might be insufficient for restoring the function of an impaired heart. Hence, various strategies have been proposed to increase the survival rate of ASCs and prompt the sustained and regulated paracrine. We summarized those strategies as follows (Fig. 3).

### Genetic modification

The modification of gene expression is a conventional approach to alter the cellular function, including promoting ASCs to release the secretome. Wang et al. [54] found that PHD2 silencing in ASCs, through the transduction with lentiviruses encoding shRNA, notably enhanced the secretion of IGF-1 and helped to reduce cardiomyocyte apoptosis through the activation of NF-γB binding to IGF-1 gene promoter. Furthermore, to improve the efficiency, Deveza et al. [55] used biodegradable polymeric nanoparticles, poly β-amino esters, as vectors to transduce the VEGF gene into ASCs. With the increased production of VEGF, ASCs could enhance HUVEC viability, migration, and tube formation. Lee’s lab [56] also developed a high-efficiency delivery system that uses poly (ethyleneglycol)-poly (amino ketal) to transfect SDF-1α into hASCs under the hypoxic condition. This method significantly enhanced the levels of SDF-1a, VEGF, and FGF-2 compared with the conventional approaches. Additionally, the genetic modification of ASCs can further enhance the therapeutic effects by increasing the expression level of desired molecules of the exosomes. For instance, after the overexpression of miR-126 in ASCs, the exosomes were more heavily loaded with miR-126, which can significantly promote microvascular generation and migration, and markedly inhibit cardiac fibrosis and inflammation [57].

### Molecular preconditioning of protein

The trophic proteins prompt ASCs to secrete paracrine factors. For instance, the growth factor, IGF-1, could facilitate ASCs releasing angiogenesis-related factors, such as VEGF-A, TGF-β, α-smooth muscle actin (α-SMA), FGF-1, MMP-2, and MMP-9 [58]. Another cytokine of platelet-derived growth factor (PDGF)-D also enhanced ASC paracrine activity, leading to the increased release of diverse growth factors including VEGF-A, FGF-1, FGF-5, leukemia inhibitory factor (LIF), and heparin binding EGF-like growth factor, as well as the strengthening of ASCs’ regenerative potential [59]. Furthermore, an apoptosis-related protein, C1q/tumor necrosis factor-related protein-9, could induce the remarkable augmentation of superoxide dismutase-3 secretion by ASCs, which was involved in the enhanced anti-apoptotic cardio-protection via N-cadherin/ERK/Nrf2 signaling pathway [60]. Specially, a kind of immunization-related protein, C-reactive protein, could promote the upregulation of VEGF-A in ASCs by activating the hypoxia-inducible factor-1α (HIF-1α) via CD64/PI3K/Akt and MAPK/ERK signaling pathways [61]. Kang et al. [38] pretreated ASCs with endothelial differentiation medium, which contained various trophic protein molecules, and found that the release of microvesicles was enhanced, and this in turn contributed to pro-angiogenesis effect after the delivery of miR-31targeting HIF-1. Consistent with Kang’s findings, Souza et al. [62] found that preconditioning of ASCs with endothelial growth medium could stimulate ASCs to secrete HGF and augment their pro-angiogenic function. Additionally, using biomaterial, the controlled release of trophic proteins allowed researchers to stimulate ASCs for a longer period of time. The bFGF retained in the gelatin hydrogel helped to promote ASCs to release HGF, VEGF, and TGF-β1 and, thus, contributed to the angiogenesis in the ischemic region [63].

### Pharmacological preconditioning

Various pharmacological molecules, including rosuvastatin [64], vitamin E [65], vitamin C [66], dimethyloxalylglycine [67], docosahexaenoic acid [68], melatonin [69], and curcumin [70], have been documented to enhance the paracrine action of ASCs and are also engaged in the anti-inflammation and anti-oxidative stress, pro-angiogenesis, promoting proliferation, and survival of implanted ASCs. Deferoxamine, an anti-oxidant compound, could stimulate ASCs to secrete the VEGF in a concentration- and time-dependent manner and thus improved the regenerative function of endothelial cells [71]. Furthermore, deferoxamine also showed a similar effect on diabetic ASCs to restore neovascularization potential through the release of paracrine factors, such as asHIF-1α, VEGF, FGF-2, and SDF-1 [72]. Regarding the treatment for MI, the sildenafil (Viagra, the inhibitor of phosphodiesterase-5)-pretreated ASCs could increase the vascular density, decrease the apoptosis and fibrosis of resident cardiomyocytes, and improve cardiac function for MI. These functions are mainly ascribed to the paracrine factors released by activated ASCs such as b-FGF, IGF, and VEGF [73]. Similarly, ASCs preconditioned with AM1241, cannabinoid receptor type II agonist, showed the inhibition of cardiac oxidative stress, apoptosis, and fibrosis. These functions also result from the enhanced release of beneficial factors such as VEGF, bFGF, HGF, and IGF-1, and the decreased level of detrimental factors such as TGF-β1 and PDGF released by ASCs through Stat3 activation via the phosphorylation of Akt and ERK1/2 [74].

### Physiological preconditioning

Hypoxia or serum-free cultivation has been favored as an effective method to stimulate the release of ASC secretome. Yang et al. [75] confirmed that hypoxia preconditioning of ASCs for 24 h led to the amplified release of HGF, IL-1, VEGF-A, FGF-2, and TGF-β, contributing to the prevention of apoptosis of cardiomyocytes through the JNK signaling pathway after MI injury. Furthermore, the capillary density in the MI region was also dramatically increased, due to the enhanced secretion of HIF-1α and VEGF after the treatment of hypoxia (2% O_2_)-preconditioned ASCs [76]. Consistent with Wang’s study, Stubbs et al. [77] further elaborated that the increased level of VEGF secreted by hypoxia-preconditioned ASCs was due to the upstream activation of HIF-1α. Interestingly, hypoxic conditioning could also improve the aged ASC secretome, which is in line with the increased level of pro-angiogenic factors such as VEGF, PGF, and HGF, and the decreased level of anti-angiogenic factors such as thrombospondin-1 and plasminogen activator inhibitor (PAI)-1 [78]. The mass spectrometry measurement demonstrated that the most regulated proteomic profiles of hypoxic-preconditioned ASCs were the ECM synthesis and cell metabolism, while several other studies showed that its therapeutic potential was mainly due to the pro-angiogenic effect [31].

In addition, the serum-deprived cultivation could also promote ASCs to change its secretome, including the release of growth factors such asIGF-1, bone morphogenetic protein (BMP)-6, FGF-9, HGF, and PDGF-D; the upregulated expressions of proprotein convertase subtilisin/kexin type (PCSK)-5; and the downregulated expressions of TGFβ1, gremlin (GREM)1, and GREM2. These molecules were responsible for ECM composition and regulating the inflammatory, fibrogenic, and angiogenic pathways [79].

### Physical preconditioning

Physical microenvironmental conditions, such as shear stress, light irradiation, and magnetic and temperature induction, have been identified to control the secretory activity of stem cells [80–84]. Bassaneze et al. [83] reported that the laminar shear stress (10 dyn/cm^2^ up to 96 h) acted on human ASCs could lead to the enhanced secretion of nitric oxide and VEGF. Another physical approach, low-level light irradiation, could also stimulate ASC spheroid to release several angiogenic factors including VEGF and bFGF and to synthesize extracellular matrix such as collagen, contributing to the increased vascular density in the ischemic vascular diseases [80]. Specially, in MI therapy, the transplantation of ASCs preloaded with superparamagnetic iron oxide (SPIO) nanoparticles, together with external application of static magnetic field (SMF), could prolong and augment the release of multiple paracrine factors such as VEGF, HGF, and IGF-1 [81]. To boost the retention of ASCs, using another electromagnetic approach, the magnetic cationic liposomes (MCLs) with a positive surface charge could be easily integrated into targeted cell membrane, and thus lead to the accumulation of MCL-labeled ASCs of a high-density level. Additionally, Ishii et al. [84] created a special multilayered ASC sheet for mouse MI therapy. This method led to the enhanced expression of VEGF and bFGF and the increase of myocardial capillaries. Nonetheless, the physical preconditioning methods that present the potential of stimulating ASCs paracrine activity are still limited.

### Cell-cell interactions

3D cell culture can effectively facilitate cell-to-cell interactions and imitate the microenvironment in vivo. These properties poise 3D cell culture as an excellent strategy to promote ASCs to release the secretome. For example, Bhang et al. [85] demonstrated that hASCs cultured as spheroids, especially with a certain critical size over 100-200 mm, exhibited the enhanced secretion of pro-angiogenic and anti-apoptotic factors such as HGF, VEGF, and FGF-2. Furthermore, they found that [47] the injection of hASC-CM from 3D culture could notably improve the blood vessel density and blood perfusion in the hindlimb ischemic diseases compared with the conventional monolayer culture, mostly attributed to the augmented cytokines in 3D culture. Additionally, to further stimulate the release of ASC secretome, Lee et al. [86] combined long-duration 3D culture with hypoxic conditioning, which led to the increase of angiogenic factors (such as VEGF, SDF, and HGF) in a time-dependent manner. Regarding the therapy for MI, transplantation of 3D cell mass (3DCMs) of ASCs could significantly increase the vasculature in the infarcted region, because of the high-level production of VEGF released by 3DCMs [87].

### Tissue engineering

Several biomaterials, such as hydrogels, self-assembling peptides (SAPs), microspheres, cell sheets, and scaffolds, can encapsulate intact cells or be captured into stem cells. This will help to achieve long-term cell retention and engraftment and thus efficiently promotes ASCs to release the secretome.

Various ASC-seeded scaffolds, including extracellular matrix scaffolds and composite scaffolds, could induce the release of angiogenic growth factors. However, the efficiency of ASC paracrine action in different scaffolds was distinct. For instance, the scaffold of small intestinal submucosa preferentially prompted ASCs to release VEGF, compared with the scaffold of a cellular dermal matrix and collagen-chondroitin sulfate-hyaluronic acid [88]. Besides the application of scaffolds, Cheng et al. [89] used a thermosensitive chitosan/gelatin hydrogel to encapsulate hASCs, which led to the enhanced concentration and sustained release of VEGF and the increased capillary density. Furthermore, another type of mixed hydrogel, containing poly (trimethylene carbonate)-*b*-poly (ethylene glycol)-*b*-poly (trimethylene carbonate) diacrylatecopolymers, combined with hypoxia conditioning, presented desirable encapsulation of hASCs and led to a more robust release of angiogenic and chemotactic factors including VEGF-A, angiopoietin-1, angiogenin, HGF, PGF, PDGF-A, leptin, SDF-1α, and MCP-1 [90]. Additionally, in MI therapy, the combination of SAPs and 3D-ASCs culture effectively increased the release of paracrine factors. The VEGF production of the combinational group within the infarcted myocardium was nearly tenfolds and twofolds greater compared with monolayer cell and 3D cell groups, respectively [91]. Another example of comprehensive tissue engineering, combined a facile cell sheet with the VEGF overexpression by genetic engineering, conferred robust and prolonged secretion of growth factors from ASCs, primarily due to the enhanced capacity of retention and survival of implanted ASCs [92].

## Application of the ASC secretome in clinical settings

Various clinical trials for cardiovascular diseases using ASCs have been conducted to demonstrate the safety, feasibility, and efficacy of cell therapy. These results confirm that the cardiac function is improved more or less, probably mediated by the paracrine effect rather than the direct cardiovascular lineage differentiation or myocardial regeneration of ASCs. The applications of ASCs in patients with cardiovascular diseases are summarized in Table 1 (registration at clinicaltrials.gov).

**Table 1:** Completed and ongoing ASC-based clinical trials for cardiovascular diseases

Clinical trial ID	Phase	Pathologies	Enrolled number	Cell delivery route	Outcome measures	Status	Study designs	Cell quantity
NCT00442806 [83]	1	STEMI	14	Intracoronary injection	Safety, MACCE, feasibility, cardiac function	Completed	Randomized, parallel assignment, double-blind	20–40 × 10^6^ cells
NCT02673164	2	Heart failure	138	Direct intra-myocardial injection	LVESV, safety	Recruiting	Randomized, parallel assignment, double-blind	100 × 10^6^ cells
NCT01449032 [86]	2	Chronic ischemic heart disease	60	Direct intra-myocardial injection	Exercise test, clinical evaluation	Completed	Randomized, parallel assignment, double-blind	72.0 ± 44.9 × 10^6^ cells
NCT02387723	1	Heart failure	10	Direct intra-myocardial injection	Safety, LVESV, LVEF, LVEDV (ml), LV end-systolic mass (g)	Completed	Single group assignment, open-label	100 × 10^6^ cells
NCT00426868 [84]	1	Ischemic heart disease	27	Direct intra-myocardial injection	Safety, MACCE, feasibility, cardiac function	Completed	Randomized, parallel assignment, double-blind	0.4–1.2 × 10^6^ cells/kg
NCT03092284	2	Heart failure	81	Direct intra-myocardial injection	LVESV, safety, LVEF, KCCQ, 6 min walking test, Seattle Angina Questionnaire	Recruiting	Randomized, parallel assignment, double-blind	100 × 10^6^ cells
NCT02052427 [85]	2	Chronic myocardial Ischemia	3	Direct intra-myocardial injection	MLHFQ, mVO2, LVESV/LVEDV, EF, perfusion defect, NYHA classification, CCS classification	Completed	Randomized, parallel assignment, double-blind	0.8 × 10^6^ cells/kg
NCT01216995	2	STEMI	23	Intracoronary injection	Infarct size, MACCE rates	Completed	Randomized, parallel assignment, double-blind	Not yet open
NCT01556022 [85]	2	Chronic ischemic heart disease	28	Direct intra-myocardial injection	Safety, SAEs, MACE, arrhythmia assessment, feasibility, cardiac function, LVESV/LVEDV, EF	Completed	Randomized, parallel assignment, double-blind	0.4 × 10^6^ cells/kg
NCT01709279	Not applicable	Ischemic heart failure	6	Intracoronary injection	All-cause harmful events	Enrolling by invitation	Single group assignment, open-label,	Not yet open

*STEMI* ST-elevation acute myocardial infarction, *LVESV* left ventricle end-systolic volume, *LVEDV* left ventricle end-diastolic volume, *MACCE* major adverse cardiac and cerebral events, *KCCQ* Kansas City Cardiomyopathy Questionnaire, *MLHFQ* Minnesota Living with Heart Failure Questionnaire, *NYHA* New York Heart Function Assessment, *CCS* Canadian Cardiovascular Society, *SAEs* serious adverse events, *MACE* major adverse cardiac events

The first clinical trial (NCT00442806) [93] in which autologous ASCs were administered to the patients with AMI was a randomized, double-blinded, parallel assignment, phase I study. This study assessed the safety and feasibility of intracoronary ASC infusion. The main endpoints included the coronary flow, major adverse cardiovascular and cerebrovascular event (MACCE), severe adverse event (SAE), congestive heart failure, LVEF, myocardial infarct size, and perfusion defect during the 6-month follow-up. The results showed that ASC infusion could significantly improve coronary perfusion defect, reduce infarct size, and enhance cardiac function. In addition to intracoronary injection, the PRECISE trial (NCT00426868) [94] reported the safety of trans-endocardial injection of autologous ASCs in patients with ischemic cardiomyopathy and displayed favorable effects in cardiac function, myocardial perfusion, and exercise capacity. Except, the ATHENA trials [95] reported another approach to transplant ASCs through direct intra-myocardial injection by electromechanical mapping-guided needle. These studies consisted of two parallel and prospective programs (ATHENA, NCT01556022; ATHENA II, NCT02052427). Thirty-one patients with an EF ≥ 20% but ≤ 45% (i.e., not eligible for revascularization) and chronic ischemic cardiomyopathy were enrolled. The SPECT results showed a trend in favor of the ASC-treated patients in terms of the differences between ASCs and placebo group for change from baseline in percent LV with stress defect. The total score of the Minnesota Living with Heart Failure Questionnaire (MLHFQ), domain scores for the SF-36, New York Heart Function Assessment (NYHA), and Canadian Cardiovascular Society (CCS) class all showed significant improvement after 12 months of post-implantation. These clinical trials have illustrated the positive efficacy of ASCs. However, the performance of ASC-based treatment did not uniformly meet anticipation. Maybe, different approaches of delivery, cell formulations, and time of ASC administration should affect engraftment efficiency and curative effect. Under the combined actions of these factors, not all output measures were positive or statistically significant in these clinical trials.

Furthermore, the MyStromalCell Trial (NCT01449032) [96] utilized a modified autologous ASCs for the therapy of chronic IHD, that were pretreated with the VEGF-A_165_. In this study, the safety of treatment was assessed. Sixty patients with significant coronary artery stenosis, an EF > 40%, and CCS/NYHA class II–III were enrolled. The results demonstrated that the intra-myocardial injection of VEGF-A_165_-stimulated ASCs was safe, and exercise capacity was significantly increased in the ASC-treated compared to the placebo group. Based on this clinical data, we speculate that the favorable outcomes may be due to the enhancement of secretome released by VEGF-A_165_-modified ASCs. The ASC modification could promote cell survival and facilitate a sustained and regulated release of paracrine factors.

As for the mechanism of cardiac repair, the paracrine effect is widely considered to be responsible for the improved cardiac function. Nevertheless, it is still difficult to directly demonstrate the involvement of factors in vivo released by ASCs in cardiac functional improvement. Although direct detecting the concentrations of paracrine factors is infeasible in the myocardial tissue in humans, their existence, to a certain extent, could be indirectly indicated by the levels in plasma. Therefore, future clinical trials should endeavor to explore the indicators for ASC-released secretome with favorable sensitivity and specificity. Moreover, it is hard to identify whether paracrine factors originate from transplanted ASCs or host cells, as it is challenging to track these soluble factors. Hence, a comparative analysis of the patient plasma before and after ASC treatment may be useful to provide some information on paracrine effect. In addition, the exact mechanisms of paracrine action have not been fully understood. The analysis of patient plasma could help to reveal the underlying mechanisms in future clinical trials.

## Conclusion

The primary mechanism of the function of ASCs in the therapy for IHD is paracrine. These factors activate local ischemic microenvironment, rescue cardiomyocytes, promote neoangiogenesis, eventually reduce infarct size, and improve cardiac function. However, the therapeutic value of ASC secretome is largely limited without long-term cell retention and engraftment after transplantation. Therefore, various strategies are proposed, including genetic, pharmacological, physiological, physical, and cytokine preconditioning, and tissue engineering, to modulate and stimulate ASCs to release the secretome. Although various effective methods help to prompt the therapeutic value of secretome, the optimal preconditioning condition and the exact triggering and regulatory mechanisms remain elusive, and the functioning proteins and RNAs in the secretome have not yet thoroughly identified. Also, it is still an open question whether the secretome therapy solely is sufficient for cardiac repair. Therefore, further studies should aim to elucidate these pivotal mechanisms and to determine better therapeutic approaches.

## Data Availability

Not applicable.

## Abbreviations

3D: Three-dimensional
3DCMs: 3D cell mass
AMI: Acute myocardial infarction
Ang: Angiopoietin
ASCs: Adipose-derived stem cells
ASC-CM: ASC-conditioned medium
ASC-exo: ASC-derived exosomes
ASK1: Apoptosis signal-regulating kinase 1
BMP: Bone morphogenetic protein
BMSCs: Bone marrow-derived mesenchymal stem cells
CCS: Canadian Cardiovascular Society
CXCL: Chemokine (C-X-C motif) ligand
ECM: Extracellular matrix
ECs: Endothelial cells
EPCs: Endothelial progenitor cells
ERK1/2/MAPK: Extracellular signal-regulated protein kinase 1/2/mitogen-activated protein kinase
ESCs: Embryonic stem cell
FGF: Fibroblast growth factor
FIH-1: Factor-inhibiting HIF-1
GREM: Gremlin
HGF: Hepatocyte growth factor
HIF-1: Hypoxia-inducible factor-1
IDO: Indoleamine 2,3-dioxygenase
IFN: Interferon
IGF: Insulin-like growth factor
IHD: Ischemic heart diseases
iPS cells: Induced pluripotent stem cells
KCCQ: Kansas City Cardiomyopathy Questionnaire
LIF: Leukemia inhibitory factor
LV: Left ventricle
LVEDV: Left ventricle end-diastolic volume
LVESV: Left ventricle end-systolic volume
MACCE: Major adverse cardiac and cerebral events
MACE: Major adverse cardiac events
MCLs: Magnetic cationic liposomes
MCP: Monocyte chemoattractant protein
MLHFQ: Minnesota Living with Heart Failure Questionnaire
MMP: Matrix metalloproteinase
MVs: Microvesicles
NGF: Nerve growth factor
NRVMs: Neonatal rat ventricular myocytes
NYHA: New York Heart Function Assessment
PAI: Plasminogen activator inhibitor
pASCs: Porcine ASCs
PDGF: Platelet-derived growth factor
PGE_2_: Prostaglandin E_2_
PGF: Placental growth factor
SAEs: Serious adverse events
SAPs: Self-assembling peptides
SDF: Stromal cell-derived factor
SMF: Static magnetic field
SPIO: Superparamagnetic iron oxide
STEMI: ST-elevation acute myocardial infarction
subASCs: ASCs derived from subcutaneous adipose
TGF: Transforming growth factor
thymASCs: ASCs derived from the thymus
TIMP: Tissue inhibitor of metalloproteinase
VEGF: Vascular endothelial growth factor
α-SMA: α-Smooth muscle actin
